# Natural radioactivity and radiological risks of common building materials used in Semnan Province dwellings, Iran

**DOI:** 10.1007/s11356-021-13469-6

**Published:** 2021-03-30

**Authors:** Morteza Imani, Mohammademad Adelikhah, Amin Shahrokhi, Ghazaleh Azimpour, Ali Yadollahi, Erika Kocsis, Edit Toth-Bodrogi, Tibor Kovács

**Affiliations:** 1 Engineering Department, Shahid Beheshti University, Tehran, Iran; 2 Institute of Radiochemistry and Radioecology, University of Pannonia, Veszprém, H-8200 Hungary; 3 Department of Natural Geography, Faculty of Geography, University of Tehran, Tehran, Iran

**Keywords:** Natural radioactivity, Building materials, Gamma-ray spectrometry, Radiological hazard, Multivariate statistical method

## Abstract

Impact assessment of building materials is a focused topic in the field of radioecology. A radiological survey has conducted to monitor radioactivity of most common building materials in Semnan Province, Iran, and assess the radiation risk. Activity concentrations of ^226^Ra, ^232^Th, and ^40^K were measured in 29 samples including nine commonly used building materials that were collected from local suppliers and manufacturers, using a high purity germanium gamma-ray detector. The activity concentrations of ^226^Ra, ^232^Th, and ^40^K varied from 6.7±1 to 43.6±9, 5.9±1 to 60±11, and 28.5±3 to 1085±113 Bq kg^−1^ with averages of 26.8±5, 22.7±4, and 322.4±4 Bq kg^−1^, respectively. By applying multivariate statistical approach (Pearson correlation, cluster, and principal component analyses (PCA)), the radiological health hazard parameters were analyzed to obtain similarities and correlations between the various samples. The Pearson correlation showed that the ^226^Ra distribution in the samples is controlled by changing the ^232^Th concentration. The variance of 95.58% obtained from PCA resulted that the main radiological health hazard parameters exist due to the concentration of ^226^Ra and ^232^Th. The resulting dendrogram of cluster analysis also shows a well coincidence with the correlation analysis.

## Introduction

Radiological hazard of building materials due to the presence of natural radioactivity has been highly investigated over the last years. The issue of radiological impact to the public plays a central role in radioecological research. Several studies have been carried out to estimate the radiological hazards and annual dose contribution of natural radioactivity in buildings. Performing a radiological impact assessment for building materials to estimate and control the radiological effects on the public and on the environment is very critical and sensitive effort due the criteria of sustainable development. The assessment of the radiological impacts should be based on measured data which data could be the input parameter for models for environmental transfer process and radiation dose assessment.

Nowadays a person spends 80% of their time indoor. This is why it is important to determine the radiation exposure what comes from the building materials. The knowledge about the concentration of the radionuclides in the building materials allows us to estimate the potential radiological hazards to inhabitants of dwellings built from such materials. Building materials contains different amounts of naturally occurring radioactive materials (NORMs), mainly radionuclides of the ^238^U and ^232^Th decay series and from the ^40^K isotope. Due to this reason, radiation protection standards have been introduced by different organizations (ICRP 1994; European commission 1999; UNSCEAR 2000, 2008; El-Taher 2010; Council of the European union 2014). The ^226^Ra, ^232^Th and ^40^K worldwide activity concentrations for soils and building materials are given in the reports of the United Nations Scientific Committee on the Effects of Atomic Radiation (UNSCEAR); these are 32, 45, 412, and Bq kg^−1^ and 50, 50, and 500 Bq kg^−1^, respectively (UNSCEAR 1993, 2008).

Population is exposed to the effects of external and internal radiation emanating from building materials. The inhalation of radon (^222^Rn), thoron (^220^Rn), and their decay products contribute to the internal exposure, while the external exposure comes from the gamma-emitting radionuclides. Radiologically the most important radionuclide is the ^226^Ra (Sas et al. 2015; Kardos et al. 2015; Kuzmanović et al. 2020; Adelikhah et al. 2021). Due to this, the aim is to minimalize the radionuclide activity concentrations in the building materials, as much as is reasonably achievable, and to minimalize the radiation dose which the public is exposed to. For this purpose, knowing the dose limits for public exposure and measuring the levels of naturally occurring background radiation emanated from the ground, air, water, food, the inside of buildings, etc. is essential for estimating the exposure of humans to natural sources of radiation (El-Taher 2010). Consequently, the definition of the dose rates helps to make some preventive measures to be sure that the doses do not exceed the recommended limits. Moreover, the knowledge about gamma radiation is important in the construction industry to be capable to adopt preventative measures and decrease the unhealthy effects of the ionizing radiation (Kovács et al. 2017a, b; Ignjatović et al. 2017; Lee et al. 2019).

Information about the radionuclide activity concentrations of building materials in Iran is limited. The knowledge of activity concentrations of the building materials is needed to assess the possible radiological hazards that the residents of buildings are exposed to. The aim of this paper is to determinate the natural radioactivity by measuring the radionuclide activity concentrations of ^226^Ra, ^232^Th, and ^40^K in 29 samples that are commonly used building materials in the construction industry in Semnan Province, Iran. The potential hazards which are associated with the studied materials were estimated by calculating the radium equivalent activity concentration, the absorbed dose, the annual effective dose, and the external and the internal hazard indexes as well as the gamma and alpha indexes that can contribute to the radiological hazards affecting population health. The results are compared with the recommended worldwide values to estimate the radiological hazard to inhabitants from the used building materials; moreover, the concentrations of radionuclides measured within this study were also compared with those from other studies. Finally, in order to determine the similarities and relationships between the various samples, the radiological data were processed by applying multivariate statistical methods including the Pearson correlation coefficient, principal component analysis (PCA), and cluster analysis (CA).

## Materials and methods

### The collection and preparation of samples

**Fig. 1:**
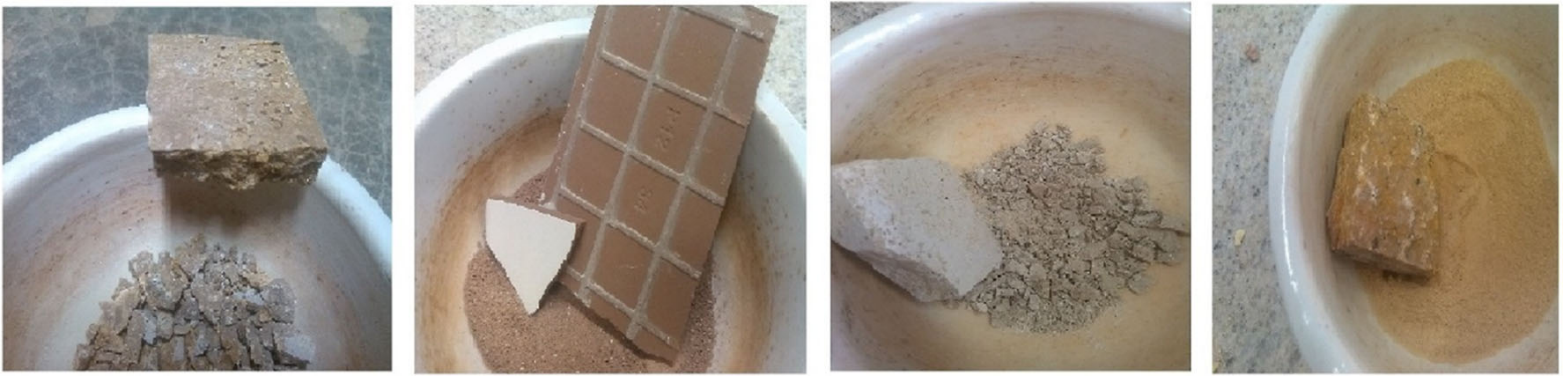
The schematic view of representative samples before and after preparation

Commonly used structural building materials, namely, sand and gravel, brick, cement, gypsum, tiles, ceramic, granite, marble, and mosaics, were collected randomly from different markets and suppliers of construction materials in Semnan Province to measure the ^226^Ra, ^232^Th, and ^40^K activity concentration (Fig. 1). All of the samples were properly cataloged, labeled, and named according to each sample’s origin and sample location. Until several days, all of the samples were dried at room temperature before being pulverized, homogenized, sieved (grain size <3 mm), and finally dried in an oven at approximately 105 °C for 24 h to constant weight. Five hundred grams of each prepared and homogenized sample were sealed in leak-proof Marinelli beakers for more than 28 days to ensure ^222^Rn and its short-lived daughter products reached equilibrium with ^226^Ra (Adelikhah et al. 2020; Shahrokhi et al. 2020).

### Radioactivity measurements

The radionuclides (^226^Ra, ^232^Th, and ^40^K) activity concentrations in the measure’s samples were determined using a semiconductor high purity germanium gamma detector. The detector was surrounded by a lead shield (10 cm thick) with electrolytic copper and internal walls composed of cadmium and coupled to a Canberra Multichannel Analyzer-Series 100. The system was calibrated according to the energy and efficiency of reference materials IAEA-375, RGU-1, RGTh-1, and RGK-1. The relative efficiency and energy resolution of the detector were 40% and 2 keV full width at half maximum from the 1332 keV peak of ^60^Co, respectively. The ^226^Ra activity concentration was determined by using the energy peaks of its decay products ^214^Pb and ^214^Bi at 352 (35%) and 609 keV (45%), respectively. The ^232^Th activity concentration was determined by the own gamma lines of its decay products ^228^Ac and ^208^Tl at 911 (28%) and 2614.51 keV (36%), respectively. The ^40^K activity concentration was calculated using its own gamma line at the energy of 1460.8 keV (11%) (Bé et al. 2007).

The activity concentration of radionuclides (Bq kg^−1^) calculated using following equation (Shahrokhi et al. 2021):

1 $$ {A}_i=\frac{1000N}{T_c{P}_{\gamma}\varepsilon M{e}^{-\lambda t}} $$

where *A*_*i*_ is activity concentration of specific radionuclide in time of sampling (Bq kg^-1^), *N* denotes the net count rate of photopeak, *T*_*c*_ represents the live counting time (second), *P*_*γ*_ stands for probability of gamma ray transition via the specific energy, *Ɛ* is the counting efficiency at specific photopeak energy, *M* is the sample mas (kg), *t* represents time interval between sampling and measuring (day), and *λ* is decay constants (expressed as $$ \raisebox{1ex}{$ Ln2$}\!\left/ \!\raisebox{-1ex}{${t}_{1/2}$}\right. $$ which *t*_*1/2*_ is half-life of radionuclide calculated). The background was measured for an empty Marinelli container with the same geometry of standard and sample container for 20,000 s. The typical measurement time for each sample was also 80,000 s.

### Multivariate statistical analysis

Multivariate statistical analysis includes the Pearson correlation coefficient as well as cluster and principal component analyses (PCA). These methods were used to clarify correlations and link between the used variables, more exactly the impact of the measured radiological parameters of measures samples and the natural radionuclides’ distribution. To evaluate the intensity of the relationship between the examined variables, the Pearson correlation coefficient was calculated. An applicable statistical method which visually presents the degree of correlations between variables is cluster analysis. PCA is used as a usual tool to summarize the set of patterns between analyzed variables in set of data. Varimax normalized method was used to process data for PCA evaluation. The main reason why PCA is commonly used is that once correlations were identified, data can be compressed reducing the number of dimensions and without any substantial information loss. To carry out the relevant statistical analysis of the obtained data, we used a software named IBM SPSS Statistics V21.0.

## Results and discussion

### Specific radioactivity

Table 1 presents the NORMs activity concentrations of the studied building materials. ^226^Ra activity concentration was found to vary from 7±1 to 44±9 with mean value of 27±5 Bq kg^−1^. The concentration of ^226^Ra was the highest in tile and the lowest in marble sample. The activity concentrations of ^232^Th in the measured samples ranged from 6±1 (in marble sample) to 60±11 Bq kg^−1^ with mean value of 23±4 Bq kg^−1^. Granite had the highest concentration of ^232^Th. The activity concentrations of ^40^K also were measured to be between 28±3 (in marble sample) and 1085±113 Bq kg^−1^ (in granite sample). The average activity concentration of ^40^K was 322±41 Bq kg^−1^. Higher activities of ^226^Ra, ^232^Th, and ^40^K were recorded in various building materials such as tiles, ceramic, bricks, and granite but, with the exception of the granite samples, did not remarkably exceed the worldwide average values.

**Table 1:** Ranged and mean values of ^226^Ra, ^232^Th, and ^40^K activity concentrations of studied building materials

Building material	No.	Activity concentration (Bq kg^−1^)			
			^226^Ra	^232^Th	^40^K
Sand and gravel	3	Range	18–31	17–25	243–454
		Average±SD	24±5	22±5	362±45
Bricks	5	Range	20–39	19–34	167–536
		Average±SD	301±7	28±4	338±54
Gypsum	3	Range	10–13	11–17	66–172
		Average±SD	12±2	14±2.0	116±14
Cement	5	Range	24–38	11–18	145–312
		Average±SD	31±6	15±4	231±31
Ceramic	3	Range	29–35	22–32	208–411
		Average±SD	32±6	27±7	292±40
Tile	3	Range	31–44	28–30	279–481
		Average±SD	36±9	29.0±7	361±41
Granite	3	Range	28–43	38–60	714–1085
		Average±SD	38±7	47±8	917±101
Marble	2	Range	7–8	6–8	28–39
		Average±SD	7±1	7±1	34±3
Mosaic	2	Range	11–19	10–11	104–187
		Average±SD	15±2	10±1	145±16
Total	29	Range	7–44	6–60	28–1085
		Average±SD	27±5	23±4	322±41
Worldwide average		Average	50^a^	50^a^	500^a^

*SD* standard deviation

^a^Worldwide average given in UNSCEAR (1993)

According to the results of Table 1, the average activity concentrations of NORMs for the mentioned building materials analyzed were, with the exception of ^40^K in granite samples, lower than the worldwide average values for these building materials of 50, 50, and 500 Bq kg^−1^, respectively (UNSCEAR 1993). Moreover, in only one granite sample, out of the 29 building materials analyzed, the activity concentration of ^232^Th exceeds the recommended value of 50 Bq kg^−1^ (UNSCEAR 1993).

**Fig. 2:**
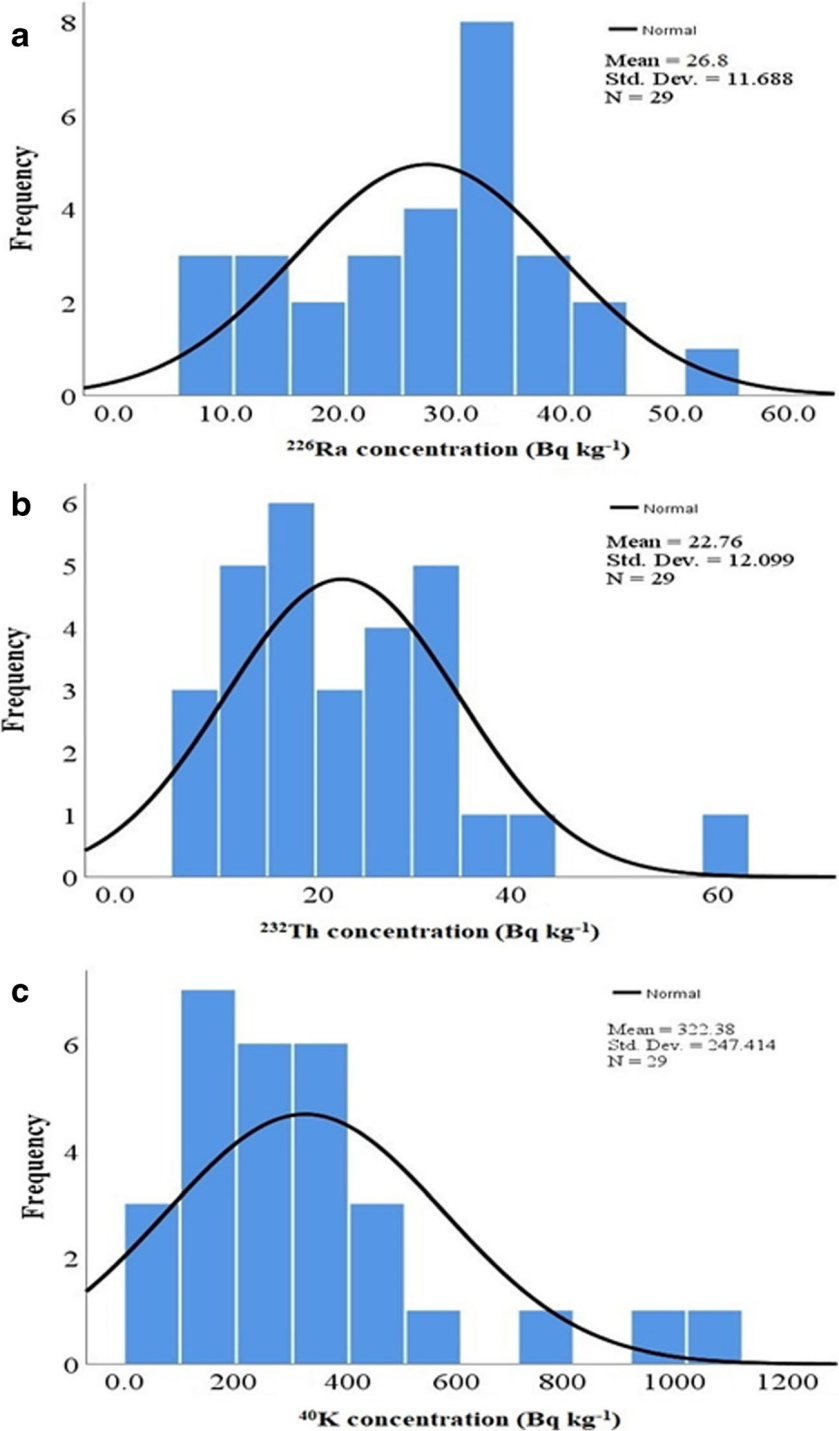
Frequency distribution of **a** ^226^Ra, **b** ^232^Th, and **c** ^40^K

The basic statistics of the studied building materials with regard to the NORMs activities are shown in Table 2. The histograms are also given in Fig. 2a–c. By analyzing the frequency distribution of all corresponded radionuclides, the histogram of ^226^Ra and ^232^Th indicates a normal distribution (bell-shaped), while ^40^K revealed in some level of multi-modality. This multimodal feature of ^40^K demonstrates the complexity of minerals in building materials.

**Table 2:** Descriptive statistics of studied building materials

Variables	^226^Ra	^232^Th	^40^K
Median (Bq kg^−1^)	30	22	279
Std. deviation (Bq kg^−1^)	11	12	247
Skewness	0	1	2
Kurtosis	-1	2	3
Geometric mean (Bq kg^−1^)	24	20	242

A comparison between the measured activity concentrations of ^226^Ra, ^232^Th, and ^40^K for the analyzed building materials and the results of similar studies reported in different countries are given in Table 3. The ranges of activity concentrations of ^226^Ra, ^232^Th, and ^40^K for samples of brick and sand recorded in this study are comparable to the values obtained in Egypt and India (Medhat 2009; Ravisankar et al. 2014), while results obtained for cement are comparable to those measured in Nigeria (Agbalagba et al. 2014). The activity concentrations of these radionuclides measured in the samples of gypsum and granite are also comparable with results from other Iranian studies (Mehdizadeh et al. 2011; Ashrafi and Jahanbakhsh 2019), while the results obtained for ceramic are comparable to Serbian (Kuzmanović et al. 2020) but dramatically lower than those from China and Saudi Arabia (Tuo et al. 2020; Al-Sewaidan 2019). The activity concentrations of these radionuclides for the samples of tile and marble were higher compared to results from Italy (Righi and Bruzzi 2006). It is noteworthy that the mean values of NORMs are changing from one location to another.

**Table 3:** A comparison between the activity concentration of radionuclides presented in studied building materials and other areas

Country	Material	No.	Activity concentration (Bq kg^−1^) (Mean)			References
			^226^Ra	^232^Th	^40^K	
China	Brick	4	14	39	678	Tuo et al. 2020
	Ceramic	6	172	135	351	
	Granite	14	356	318	1636	
	Concrete	4	16	51	605	
Serbia	Brick	12	45	49	646	Kuzmanović et al. 2020
	Ceramic tile	5	67	61	828	
	Concrete tile	2	30	18	255	
	Granite	2	200	77	1280	
	Concrete	10	17	21	253	
Iran	Granite	48	66	57	996	Ashrafi and Jahanbakhsh 2019
	Granite	29	77	45	1017	Abbasi 2013
	Gravel	32	20	6	451	Mehdizadeh et al. 2011
	Brick	77	37	12	851	
	Gypsum	30	8	2	116	
	Cement	17	40	29	291	
Egypt	Sand	15	33	27	385	Medhat 2009
	Gravel	18	10	5	159	
	Brick	30	30	21	289	
	Gypsum	21	39	25	226	
	Ceramics	29	52	33	450	
	Tile	30	35	23	377	
	Granite	16	65	60	920	
	Marble	25	32	25	466	
Italy	Brick	7	58	51	473	Righi and Bruzzi 2006
	Ceramics	2	52	42	450	
	Tile	3	20	25	427	
	Granite	6	81	129	1065	
	Concrete	2	15	16	310	
	Marble	1	1	4	20	
Saudi Arabia	Ceramic	20	89	105	773	Al-Sewaidan 2019
	Cement	4	22	10	102	
Qatar	Sand	4	13	3	225	Al-Sulaiti et al. 2011
	Cement	6	23	10	120	
	White cement	3	19	5	63	
Nigeria	Cement	5	30	25	251	Agbalagba et al. 2014
	White cement	6	42	30	340	
India	Sand	7	11	130	297	Ravisankar et al. 2014
	Brick	8	5	23	374	
	Cement	3	37	34	188	
Iran	Sand	3	24	22	362	Current study
	Brick	5	31	28	338	
	Gypsum	3	12	14	116	
	Cement	5	31	15	231	
	Ceramic	3	32	27	292	
	Tile	3	36	29	361	
	Granite	3	38	47	917	
	Marble	2	7	7	34	
	Mosaic	2	15	10	146	

### Evaluation of radiological hazard effects

Several radiological parameters, such as radium equivalent activity, external and internal hazard index, absorbed dose rate, external gamma radiation, and internal alpha radiation, were calculated to evaluate the potential radiological hazards and assess the radiation risk to human.

#### Radium equivalent activity

Since NORMs are composed of different amounts of ^226^Ra, ^232^Th, and ^40^K, the radium equivalent activity (Ra_eq_) index was used. It is calculated according to the assumption that 370 Bq kg^−1^ for ^226^Ra, 259 Bq kg^−1^ for ^232^Th, and 4810 Bq kg^−1^ for ^40^K produce the same gamma radiation dose and can be calculated using Eq. 2 (Beretka and Mathew 1985):

2 $$ {\mathrm{Ra}}_{\left(\mathrm{Eq}\right)}={A}_{\left(226\mathrm{Ra}\right)}+\left({A}_{\left(232\mathrm{Th}\right)}\times 1.430\right)+\left({A}_{\left(40\mathrm{K}\right)}\times 0.077\right) $$

where *A*_Ra_, *A*_Th_, and *A*_K_ are the activity concentrations of ^226^Ra, ^232^Th, and ^40^K, respectively, in Bq kg^−1^. The recommended maximum value of the radium equivalent activity (Ra_eq_) index for the given building material should not exceed 370 Bq kg^−1^, corresponding to the annual effective dose of 1.5 mSv y^−1^ (NEA-OECD 1979). Table 4 contains the range and mean values of the radium equivalent activities of each studied building material. Accordingly, Ra_eq_ in all samples varied from 18 to 184 Bq kg^−1^ with a mean value of 84 Bq kg^−1^. The highest value of Ra_eq_ is estimated in samples of granite (184 Bq kg^−1^), which is significantly lower than 370 Bq kg^−1^.

**Table 4:** Results of different hazard indices associated with the radioactivity of the studied building materials

Building material	No.		Ra_eq_ (Bq kg^−1^)	D (nGy h^−1^)	AED (μSv y^−1^)	*I*_γ_	*I*_α_	*H*_ex_	*H*_in_
Sand and gravel	3	Range	76–89	71–80	348–393	0.29–0.33	0.09–0.15	0.21–0.24	0.25–0.31
		Average±SD	83±16	75±7	366±37	0.31±0.06	0.12±0.03	0.22±0.04	0.29±0.06
Bricks	5	Range	67–108	59–96	287–469	0.24–0.39	0.10–0.20	0.18–0.29	0.25–0.40
		Average±SD	96±17	86±8	421±38	0.35±0.06	0.15±0.03	0.26±0.05	0.34±0.06
Gypsum	3	Range	38–42	34–36	169–179	0.14–0.15	0.05–0.07	0.10–0.11	0.13–0.15
		Average±SD	41±6	35±3	174±13	0.15±0.02	0.06±0.01	0.11±0.02	0.14±0.02
Cement	5	Range	64–76	57–68	281–333	0.23–0.27	0.12–0.19	0.17–0.20	0.24–0.31
		Average±SD	70±14	63±6	309±32	0.25±0.05	0.15±0.03	0.19±0.04	0.27±0.05
Ceramic	3	Range	82–107	73–95	356–466	0.29–0.33	0.15–0.17	0.22–0.29	0.32–0.37
		Average±SD	93±20	83±9	406±44	0.34±0.07	0.16±0.03	0.25±0.05	0.34±0.07
Tile	3	Range	100–108	88–97	434–478	0.36–0.40	0.15–0.22	0.27–0.29	0.36–0.41
		Average±SD	105±22	94±10	460±50	0.39±0.08	0.18±0.04	0.28±0.06	0.38±0.08
Granite	3	Range	163–184	150–168	735–826	0.63–0.70	0.14–0.22	0.44–0.50	0.52–0.61
		Average±SD	176±26	160±12	786±60	0.67±0.1	0.19±0.03	0.48±0.07	0.58±0.09
Marble	2	Range	18–21	16–18	77–89	0.06–0.07	0.03–0.04	0.05–0.06	0.07–0.08
		Average±SD	20±3	17±1	83±7	0.07±0.01	0.04±0.01	0.05±0.01	0.07±0.01
Mosaic	2	Range	37–42	36–37	177–182	0.14–0.15	0.06–009	0.10–0.11	0.14–0.16
		Average±SD	41±6	33±3	180±13	0.15±0.02	0.07±0.01	0.11±0.02	0.15±0.02
Total	29	Range	18–184	16–168	77–826	0.06–0.70	0.03–0.22	0.05–0.50	0.07–0.61
		Average±SD	84±15	76±7	371±35	0.31±0.1	0.13±0.03	0.23±0.04	0.30±0.06
Recommended value or worldwide average			370^a^	50^b^	1000^c^	≤1^d^	≤1^e^	≤1^f^	≤1^g^

^a^Any radium equivalent activity concentration over this value may raise radiation hazard according to NEA-OECD (1979)

^b^Worldwide average background gamma radiation reported by UNSCEAR (2008)

^c^The worldwide average value of AED reported by UNSCEAR (2008)

^d^The reference level radioactivity index in building material according to UNSCEAR (2000)

^e^The recommended value reported in ICRP (1994))

^f^The recommended value reported in Beretka and Matthew (1985)

^g^The recommended value reported in Krieger (1981)

#### Absorbed gamma dose rate and the annual effective dose rate

The absorbed gamma dose rate, caused by NORMs in building materials, and the corresponding annual effective dose rate were calculated using Eqs. 3 and 4, respectively (European Commission 1999):

3 $$ D=\left(0.92\times {A}_{\left(226\mathrm{Ra}\right)}\right)+\left(1.1\times {A}_{\left(232\mathrm{Th}\right)}\right)+\left(0.080\times {A}_{\left(40\mathrm{K}\right)}\right) $$

4 $$ {\mathrm{AED}}_{\left(\mathrm{indoor}\right)}=D\times T\times 0.8\times 0.7\times {10}^{-3} $$

where *D* represents the absorbed gamma dose rate from NORMs (nGy h^−1^), *AED* stands for the annual effective dose rate (μSv y^−1^), 0.7 is the conversion factor (Sv Gy^−1^), 0.8 is the indoor occupancy factor, and *T* denotes the number of hours in a year (8760 h y^−1^).

The estimated absorbed gamma dose rate in air (D) and annual effective dose rate (AED) of different types of structural building materials are also presented in the fifth and sixth columns of Table 4. From the data in Table 4, the estimated maximum absorbed gamma dose rate of 168 nGy h^−1^ was in samples of granite, while the minimum value of approximately 16 nGy h^−1^ was recorded in samples of marble. The estimated mean value of D in the studied samples is 75.58 nGy h^−1^, which is slightly higher than the worldwide average provided by the European Commission in 1999. The values of AED vary from 77 for marble to 826 μSv y^−1^ for granite. The estimated mean value of AED for all samples is 371 μSv y^−1^, which is less than the average value for all the building materials.

**Fig. 3:**
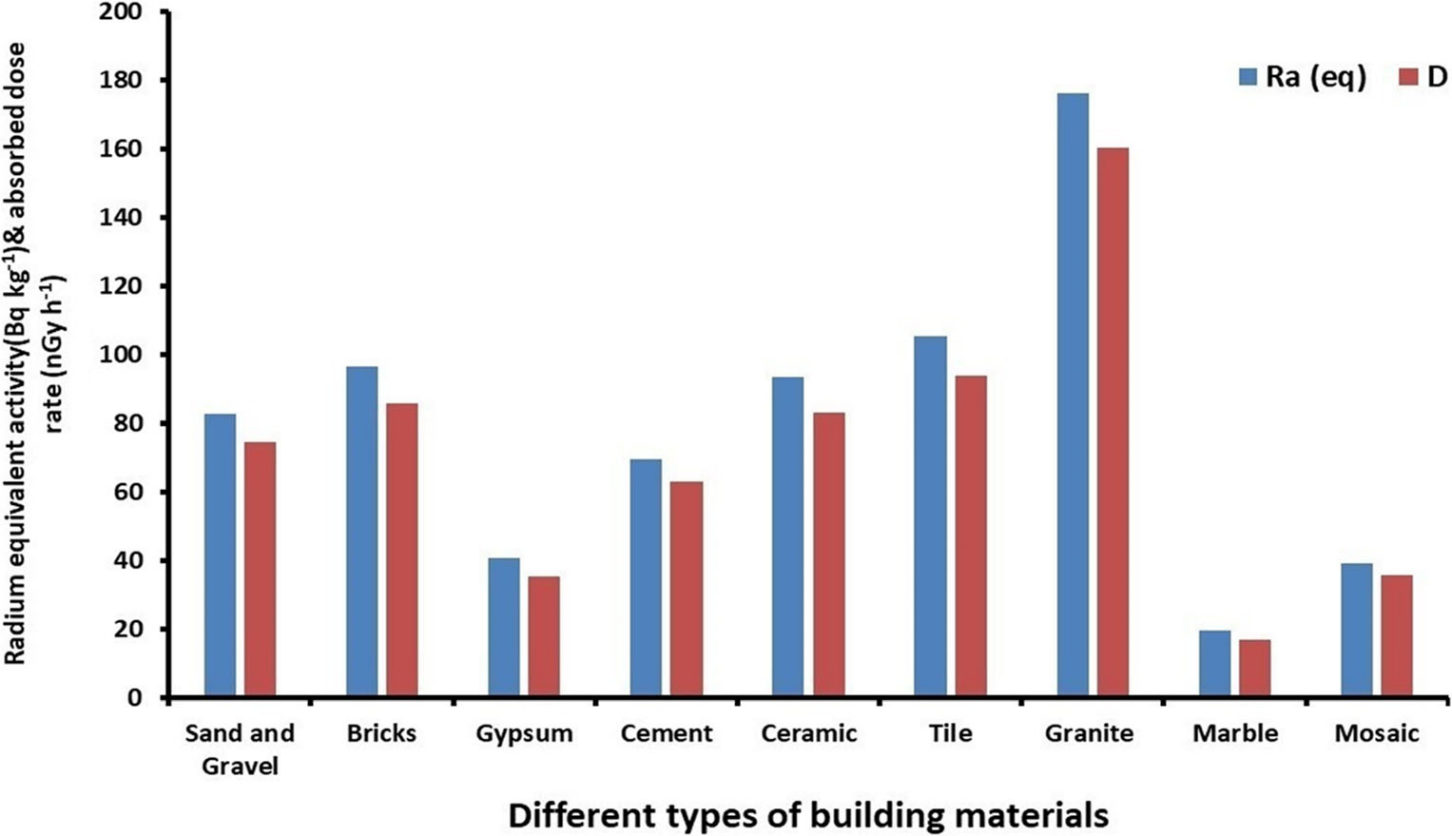
Mean values of Ra_eq_ and D of studied building materials

The mean values of Ra_eq_ and D of studied building materials are also shown in Fig. 3.

#### Hazard indices for external gamma radiation (*H*_ex_, *I*_γ_)

In this paper, the following hazard index for the external gamma radiation doses emitted from building materials to ensure their safe use was applied (Beretka and Matthew 1985):

5 $$ {H}_{\mathrm{ex}}=\left({A}_{\left(226\mathrm{Ra}\right)}/370\right)+\left({A}_{\left(232\mathrm{Th}\right)}/259\right)+\left({A}_{\left(40\mathrm{K}\right)}/4810\right)\le 1 $$

where *A* denotes the activity concentrations of specific radionuclides (Bq kg^−1^). *H*_ex_ = 1 corresponds to a radium equivalent activity of 370 Bq kg^−1^. If the limit is exceeded (*H*_ex_ > 1), it means the potential external dose to exposed individual would be higher than the acceptable level meaning a potential health risk to the public. The European Commission (EC) also proposed an index called the gamma index (*I*_γ_) to verify whether the guidelines issued by the EC concerning the usage of building materials are met. *I*_γ_ is calculated using the following formula (European Commission 1999):

6 $$ {I}_{\upgamma}=\left({A}_{\left(226\mathrm{Ra}\right)}/300\right)+\left({A}_{\left(232\mathrm{Th}\right)}/200\right)+\left({A}_{\left(40\mathrm{K}\right)}/3000\right)\le 1 $$

**Fig. 4:**
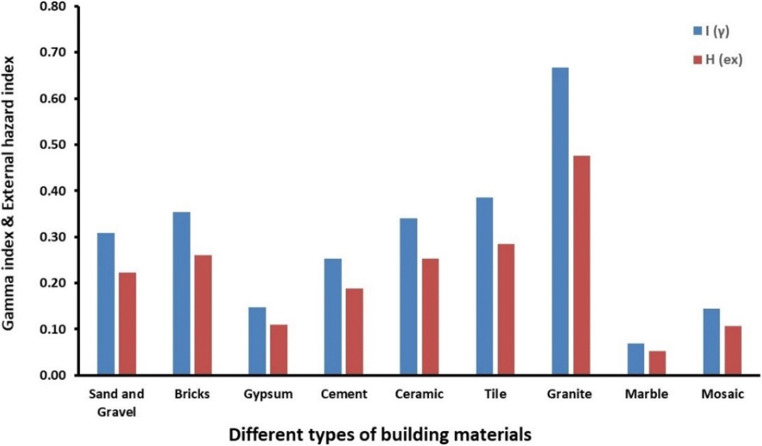
Mean values of the gamma index and external hazard index of building materials from Semnan Province, Iran

The highest values of *H*_ex_ and *I*_γ_, which were both recorded in granite, are 0.50 and 0.70, respectively (see Table 4). Moreover, the obtained corresponding mean values were both below the recommended level of 1 (Beretka and Matthew 1985; UNSCEAR 2000). Therefore, we can conclude that the external gamma radiation does not pose any radiological hazards when these building materials are used for construction in Semnan Province, Iran. A comparison between the two indices for all types of building materials is shown in Fig. 4.

#### Hazard indices for internal alpha radiation (*H*_in_, *I*_α_)

In order to measure the excess internal alpha radiation exposure caused by inhalation of ^222^Rn and its short-lived decay products originating from building materials, the internal hazard index (*H*_in_) can be used, which has been defined as (Beretka and Mathew 1985; El-Taher 2010):

7 $$ {H}_{\mathrm{in}}=\left({A}_{\left(226\mathrm{Ra}\right)}/185\right)+\left({A}_{\left(232\mathrm{Th}\right)}/259\right)+\left({A}_{\left(40\mathrm{K}\right)}/4810\right)\le 1 $$

*H*_in_ should be less than 1 for the use of building materials in the construction of dwellings to be regarded as safe (Krieger 1981). Alpha index (*I*_α_) was also suggested by Krieger and Stoulos as given below (Krieger 1981; Stoulos et al. 2003):

8 $$ {I}_{\upalpha}={A}_{\left(226\mathrm{Ra}\right)}/200 $$

**Fig. 5:**
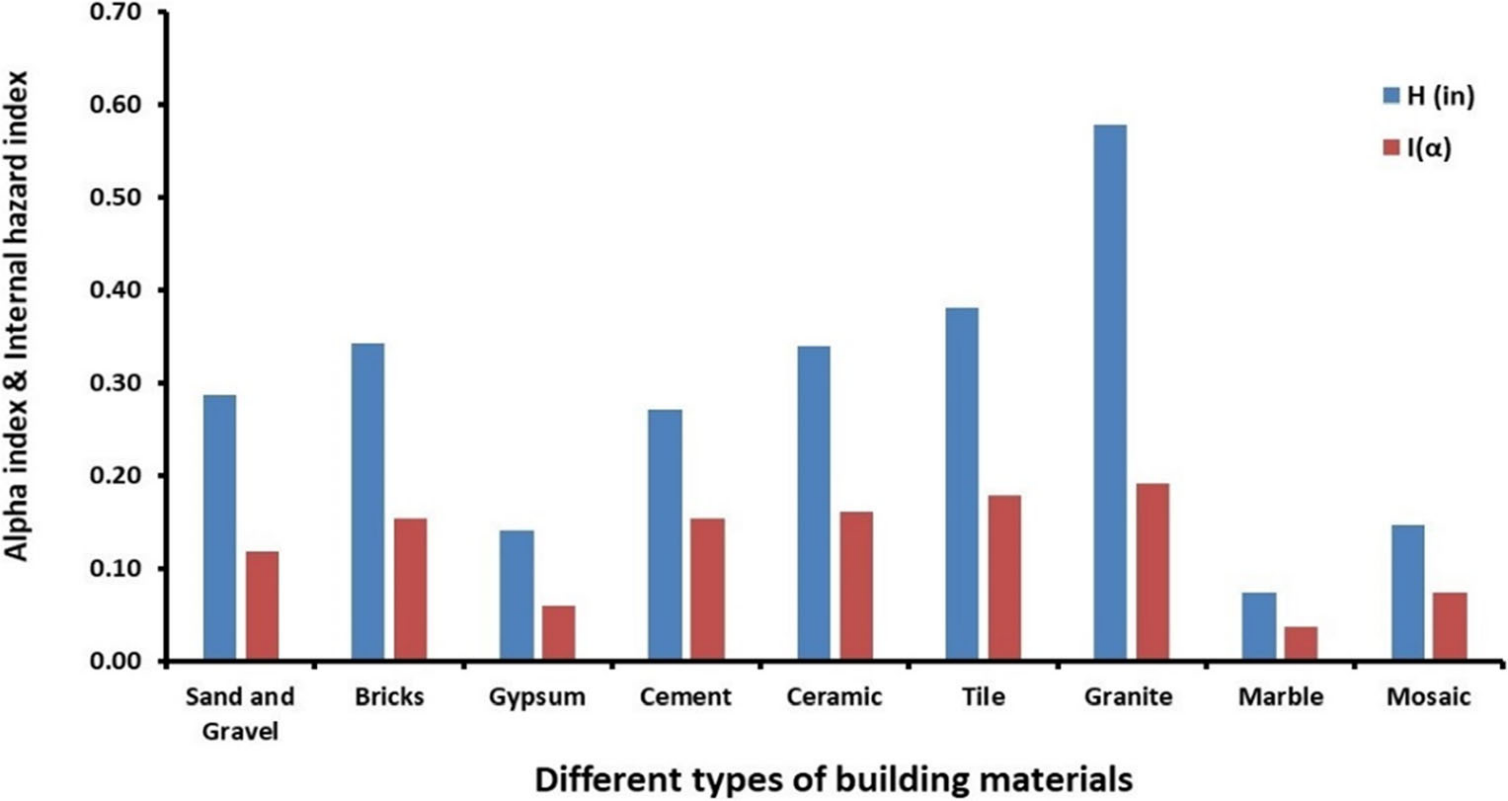
Mean values of the *I*_α_ and *H*_in_ of considered building materials

According to Table 4, the highest values of *H*_in_ and *I*_α_ are 0.61 (in granite) and 0.22 (in tile and granite), respectively, revealing that the internal hazard is below the critical value. Figure 5 also illustrates the mean values of both hazard indices with regard to the internal alpha radiation and considered samples.

## Multivariate statistical analysis

### Analysis of Pearson’s correlation coefficient

In order to determine the relation and strength of association between several radiological parameters and radionuclides, Pearson correlation analysis has been done. The data from Table 5 (The Pearson coefficient analyses) indicates a strong positive and statistically significant correlation between ^226^Ra and ^232^Th because the decay series of radium and thorium appear together in nature (Tanasković et al. 2012). Moreover, there is a direct correlation among ^226^Ra and ^232^Th with all of the calculated radiological parameters possibly because the building materials are rich in ^226^Ra and ^232^Th, which plays a significant role in assessing the hazardous nature of such materials. Furthermore, a weaker correlation, albeit still statistically significant, was observed between these two radionuclides and ^40^K.

**Table 5:** Pearson’s correlation matrix for variables

Parameters	^226^Ra	^232^Th	^40^K	*I*_γ_	Ra_eq_	D	AED	*H*_ex_	*H*_in_	*I*_α_
^226^Ra	1									
^232^Th	0.813	1								
^40^K	0.467	0.689	1							
*I*_γ_	0.838	0.946	0.792	1						
Ra_eq_	0.852	0.952	0.776	0.998	1					
D	0.844	0.944	0.788	1.00	0.999	1				
AED	0.844	0.944	0.788	1.00	0.999	1.00	1			
*H*_ex_	0.852	0.952	0.776	0.999	1.00	0.999	0.999	1		
*H*_in_	0.899	0.942	0.733	0.984	0.991	0.988	0.988	0.990	1	
*I*_α_	0.999	0.774	0.484	0.787	0.812	0.799	0.799	0.809	0.881	1

### Principal component analysis

**Fig. 6:**
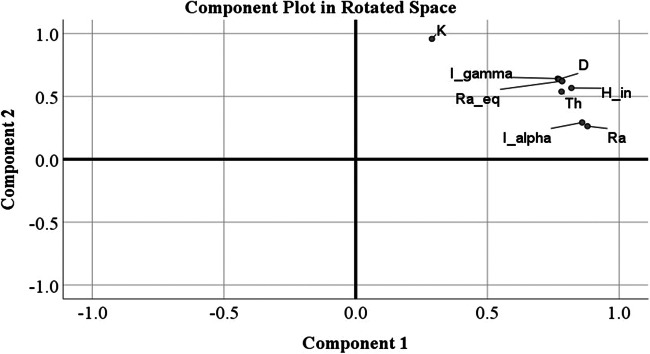
Graphical representation of factors 1 and 2

The principal component analysis (also known as factor analysis) is multivariate statistical technique used to identify important components that explain most of the variances of the original system. It is designed to reduce a set of original variables to a small number of indices for analyzing similarities and differences present among the observed variables that are not readily evident from simple correlation analysis. In this study, the varimax rotation was applied with Kaiser normalization procedure to process the PCA among the radiological parameters (Kaiser 1958). From the correlation matrix, to describe the number of notable factors the eigenvalues and eigenvectors are extracted (only factors with eigenvalues greater than 1) and explained the percent of variance. The values of rotated factors 1 and 2 are reported in Table 6. Factor analysis (FA) yielded two factors with eigenvalues < 1, explaining 95.58% of the total variance. From the rotated spaces of components 1 and 2 (Fig. 6), factor 1 accounts for 59.83% of the total variance, identified mostly by high positive loading of ^232^Th and ^226^Ra activity concentrations. Factor 2 accounts for 35.75% of the total variance and related to significant positive loading of ^40^K. From the overall FA, it can be concluded that ^226^Ra and ^232^Th mostly enhance the radioactivity of all the building materials.

**Table 6:** Rotated factor loadings of variables

Variables	Component	
	1	2
^226^Ra	0.88	0.26
^232^Th	0.78	0.54
^40^K	0.29	0.96
*I*_γ_	0.77	0.64
Ra(eq)	0.78	0.62
D	0.77	0.64
AED	0.77	0.64
H(ex)	0.78	0.62
H(in)	0.82	0.57
*I*_α_	0.86	0.29
% of variance explained	59.83	35.75

### Cluster analysis

**Fig. 7:**
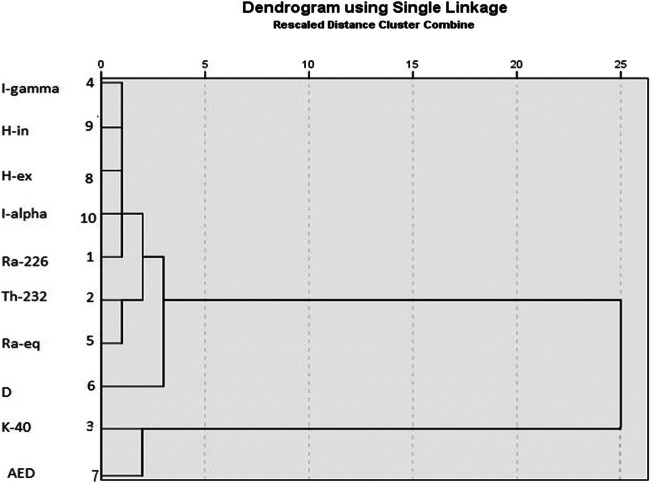
Dendrogram showing the clustering of variables

CA is a data classification technique that is comprised of a series of multivariate methods, which are used to identify true groups of data. The degree of similarity between the radioactivity and calculated radiological hazard parameters was determined by CA using IBM SPSS Statistics version 21 software. In CA, the linkage method was used to find out the correlation coefficient distance between radiological parameters, the outcome dendrogram shown in Fig. 7. In this dendrogram, all 10 parameters can be classified into three major clusters. Cluster I consists of ^226^Ra, ^232^Th, AED, *H*_ex_, *H*_in_, *I*_α_, and *I*_γ_. The results from this cluster reveal that the main radiological health hazard parameters exist due to the ^226^Ra and ^232^Th activity concentrations; while cluster II is associated with Ra_eq_ and D; and cluster III includes ^40^K, which indicates that the activity concentration of ^40^K does not contribute significantly to the natural radioactivity. The results according to the derived dendrogram are in good agreement with correlation and factor analyses. Therefore, the data belongs to studied parameters primarily relies on the natural radioisotope’s activity concentrations.

## Conclusion

Since gamma-rays emitted from building material can easily travel long distances within the surrounding environment, human beings may continuously expose by gamma radiation and adverse health effects may occurred via extended period of exposure. In this paper, the gamma spectrometric analysis of 29 common building materials, divided into 9 groups, used to construct buildings in Semnan Province, Iran, was performed. The activity concentrations of ^226^Ra and ^232^Th in the measured samples were found to vary from 7±1 to 44±9 and 6±1 to 60±11 Bq kg^−1^ with mean values of 27±5 and 23±4 Bq kg^−1^, respectively. The activity concentration of ^40^K also was measured to be between 28±3 and 1085±113 Bq kg^−1^ with average value of 322±41 Bq kg^−1^.

Based on the activity concentrations measured, the representative gamma index, absorbed dose rate, and annual effective dose find great significance to understand the health hazards from gamma-radiation exposures. The potential radiological hazards associated with the studied materials were estimated. Accordingly, Ra_eq_ in all samples varied from 18 to 184 Bq kg^−1^ with a mean value of 84 Bq kg^−1^. The estimated mean value of D and AED for all samples is 76 nGy h^−1^ and 371 μSv y^−1^, respectively. In case of *H*_ex_ and *I*_γ_, the obtained corresponding mean values were 0.23 and 0.31, respectively. Both hazard indices with regard to the internal alpha radiation (*H*_in_ and *I*_α_), were also calculated to be 0.3 and 0.13, respectively. Consequently, it can be concluded that most results fell below the average values for building materials worldwide, therefore, in terms of their inhabitants, buildings constructed from such materials are radiologically safe. The results draw attention to the use of granite, brick, ceramic, and tile in the construction of dwellings.

The results of the multivariate statistical approach, in order to get a well-founded conclusion with regard to the distribution of radioactive elements in the building materials studied, suggest that the calculated radiological parameters are mostly due to the activity concentrations of ^226^Ra and ^232^Th.

## Data Availability

All data generated or analyzed during this study are included in this published article anyway datasets are available from the corresponding author on reasonable request.

## References

[CR1] Abbasi A (2013). Calculation of gamma radiation dose rate and radon concentration due to granites used as building materials in Iran. Radiat Prot Dosim.

[CR2] Adelikhah M, Shahrokhi A, Chalupnik S, Toth-Bodrogi E, Kovács T (2020). High level of natural ionizing radiation at a thermal bath in Dehloran, Iran. Heliyon.

[CR3] Adelikhah M, Shahrokhi A, Imani M, Chalupnik S, Kovács T (2021). Radiological assessment of indoor radon and thoron concentrations and indoor radon map of dwellings in Mashhad, Iran. Int J Environ Res Public Health.

[CR4] Agbalagba EO, Osakwe ROA, Olarinoye IO (2014). Comparative assessment of natural radionuclide content of cement brands used within Nigeria and some countries in the world. J Geochem Explor.

[CR5] Al-Sewaidan HA (2019). Natural radioactivity measurements and dose rate assessment of selected ceramic and cement types used in Riyadh, Saudi Arabia. J King Saud Univ Sci.

[CR6] Al-Sulaiti H, Alkhomashi N, Al-Dahan N, Al-Dosari M, Bradley DA, Bukhari S, Matthews M, Regan PH, Santawamaitre T (2011). Determination of the natural radioactivity in Qatarian building materials using high-resolution gamma-ray spectrometry. Nucl Instrum Meth A.

[CR7] Ashrafi S, Jahanbakhsh O (2019). Measurement of natural radioactivity of Iranian granite samples using beta–gamma coincidence spectrometer and maximum likelihood method. Environ Earth Sci.

[CR8] Bé MM, Chechev VP, Khlopin VG (2007). Update of X ray and gamma ray decay data standards for detector calibration and other applications, Volume 2: Data Selection Assessment and Evaluation Procedures.

[CR9] Beretka J, Matthew PJ (1985). Natural radioactivity of Australian building materials, industrial wastes and by-products. Health Phys.

[CR10] Council Directive 2013/59/Euratom of 5 Dec. 2013 (2014) Laying down basic safety standards for protection against the dangers arising from exposure to ionising radiation, and repealing directives 89/618/Euratom, 90/641/Euratom, 96/29/Euratom, 97/43/ Euratom and 2003/122/Euratom. L13, vol 57. ISSN 1977-0677.https ://ec.europ a.eu/energ y/sites /ener/files /docum ents/CELEX -32013 L0059 -EN-TXT.pdf

[CR11] El-Taher A (2010). Gamma spectroscopic analysis and associated radiation hazards of building materials used in Egypt. Radiat Prot Dosim.

[CR12] EUROPEAN COMMISSION (1999). Radiological protection principles concerning the natural radioactivity of building materials, Radiation Protection Report - RP-112.

[CR13] ICRP(International Commission on Radiological Protection) (1994) Protection Against ^222^Rn at home and at work. ICRP Publication-65. Pergamon Press, Oxford.

[CR14] Ignjatović I, Sas Z, Dragaš J, Somlai J, Kovács T (2017). Radiological and material characterization of high-volume fly ash concrete. J Environ Radioact.

[CR15] Kaiser HF (1958). The varimax criterion for analytic rotation in factor analysis. Psychometrika.

[CR16] Kardos R, Sas Z, Hegedűs M, Shahrokhi A, Somlai J, Kovacs T (2015). Radionuclide content of NORM by-products originating from the coal-fired power plant in Oroszlány (Hungary). Radiat Prot Dosim.

[CR17] Kovács T, Bátor G, Schroeyers W, Labrincha J, Puertas F, Hegedus M, Nicolaides D, Sanjuán MA, Krivenko P, Grubeša IN, Sas Z (2017a) From raw materials to NORM by-products. In: Naturally Occurring Radioactive Materials in Construction. Woodhead Publishing, pp. 135-182.

[CR18] Kovács T, Shahrokhi A, Sas Z, Vigh T, Somlai J (2017). Radon exhalation study of manganese clay residue and usability in brick production. J Environ Radioact.

[CR19] Krieger R (1981). Radioactivity of construction materials. Betonw Fert Tech.

[CR20] Kuzmanović P, Todorović N, Filipović Petrović L, Mrđa D, Forkapić S, Nikolov J, Knežević J (2020). Radioactivity of building materials in Serbia and assessment of radiological hazard of gamma radiation and radon exhalation. J Radioanal Nucl Chem.

[CR21] Lee KY, Hwang S, Kim Y, Ko KS (2019). Measurement of NORM in geologic and building materials by pair measurement–gamma spectrometry. J Radioanal Nucl Chem.

[CR22] Medhat ME (2009). Assessment of radiation hazards due to natural radioactivity in some building materials used in Egyptian dwellings. Radiat Prot Dosim.

[CR23] Mehdizadeh S, Faghihi R, Sina S (2011). Natural radioactivity in building materials in Iran. Nukleonika.

[CR24] NEA-OECD (Organization for Economic Co-operation and Development) (1979) Exposure to radiation from radioactivity in building materials. Report by a Group of Experts of The OECD Nuclear Energy Agency.

[CR25] Ravisankar R, Vanasundari K, Suganya M, Raghu Y, Rajalakshmi A, Chandrasekaran A, Sivakumar S, Chandramohan J, Vijayagopal P, Venkatraman B (2014). Multivariate statistical analysis of radiological data of building materials used in Tiruvannamalai, Tamilnadu, India. Appl Radiat Isot.

[CR26] Righi S, Bruzzi L (2006). Natural radioactivity and radon exhalation in building materials used in Italian dwellings. J Environ Radioact.

[CR27] Sas Z, Somlai J, Szeiler G, Kovács T (2015). Usability of clay mixed red mud in Hungarian building material production industry. J Radioanal Nucl Chem.

[CR28] Shahrokhi A, Adelikhah M, Chalupnik S, Kocsis E, Toth-Bodrogi E, Kovács T (2020). Radioactivity of building materials in Mahallat, Iran – an area exposed to a high level of natural background radiation – attenuation of external radiation doses. Mater Constr.

[CR29] Shahrokhi A, Adelikhah M, Chalupnik S, Kovács T (2021) Multivariate statistical approach on distribution of natural and anthropogenic radionuclides and associated radiation indices along the north-western coastline of Aegean Sea, Greece. Mar Pollut Bull 163:112009. 10.1016/j.marpolbul.2021.112009 10.1016/j.marpolbul.2021.112009 33477060

[CR30] Stoulos S, Manolopoulou M, Papastefanou C (2003). Assessment of natural radiation exposure and radon exhalation from building materials in Greece. J Environ Radioact.

[CR31] Tanasković I, Golobocanin D, Miljević N (2012). Multivariate statistical analysis of hydrochemical and radiological data of Serbian spa waters. J Geochem Explor.

[CR32] Tuo F, Peng X, Zhou Q, Zhang J (2020). Assessment of natural radioactivity levels and radiological hazards in building materials. Radiat Prot Dosim.

[CR33] UNSCEAR (1993) Sources, effects and risks of ionizing radiation. United Nations Scientific Committee on the Effects of Atomic Radiation, United Nations New York

[CR34] UNSCEAR (2000) Sources and effects of ionizing radiation. United Nations Scientific Committee on Effects of Atomic Radiation. Exposures from Natural Radiation Sources, Annex B. United Nations Publication, New York, USA

[CR35] UNSCEAR (2008). Effects of ionizing radiation: report to the General Assembly with scientific annexes, United Nations Scientific Committee on Effects of Atomic Radiation.

